# Dysbiosis of the Fecal Microbiota in Cattle Infected with *Mycobacterium avium* subsp. *paratuberculosis*

**DOI:** 10.1371/journal.pone.0160353

**Published:** 2016-08-05

**Authors:** Marie-Eve Fecteau, Dipti W. Pitta, Bonnie Vecchiarelli, Nagaraju Indugu, Sanjay Kumar, Susan C. Gallagher, Terry L. Fyock, Raymond W. Sweeney

**Affiliations:** Department of Clinical Studies-New Bolton Center, School of Veterinary Medicine, University of Pennsylvania, Kennett Square, Pennsylvania, United States of America; Universita di Sassari, ITALY

## Abstract

Johne's disease (JD) is a chronic, intestinal infection of cattle, caused by *Mycobacterium avium* subsp. *paratuberculosis* (MAP). It results in granulomatous inflammation of the intestinal lining, leading to malabsorption, diarrhea, and weight loss. Crohn’s disease (CD), a chronic, inflammatory gastrointestinal disease of humans, has many clinical and pathologic similarities to JD. Dysbiosis of the enteric microbiota has been demonstrated in CD patients. It is speculated that this dysbiosis may contribute to the intestinal inflammation observed in those patients. The purpose of this study was to investigate the diversity patterns of fecal bacterial populations in cattle infected with MAP, compared to those of uninfected control cattle, using phylogenomic analysis. Fecal samples were selected to include samples from 20 MAP-positive cows; 25 MAP-negative herdmates; and 25 MAP-negative cows from a MAP-free herd. The genomic DNA was extracted; PCR amplified sequenced on a 454 Roche platform, and analyzed using QIIME. Approximately 199,077 reads were analyzed from 70 bacterial communities (average of 2,843 reads/sample). The composition of bacterial communities differed between the 3 treatment groups (P < 0.001; Permanova test). Taxonomic assignment of the operational taxonomic units (OTUs) identified 17 bacterial phyla across all samples. *Bacteroidetes* and *Firmicutes* constituted more than 95% of the bacterial population in the negative and exposed groups. In the positive group, lineages of *Actinobacteria* and *Proteobacteria* increased and those of *Bacteroidetes* and *Firmicutes* decreased (P < 0.001). *Actinobacteria* was highly abundant (30% of the total bacteria) in the positive group compared to exposed and negative groups (0.1–0.2%). Notably, the genus *Arthrobacter* was found to predominate *Actinobacteria* in the positive group. This study indicates that MAP-infected cattle have a different composition of their fecal microbiota than MAP-negative cattle.

## Introduction

Johne's disease (JD), also known as paratuberculosis, is a chronic, incurable, gastrointestinal infection of cattle and other domestic and wild ruminants, caused by *Mycobacterium avium* subsp. *paratuberculosis* (MAP). The infection results in granulomatous inflammation of the intestinal lining, leading to malabsorption, chronic diarrhea, and weight loss in clinically affected animals. Most animals become infected during the neonatal period, but due to MAP’s long incubation period, clinical signs usually develop much later (2–5 years post-infection) [1]. Although vaccination has been found to reduce the incidence of clinical disease, it is not fully protective [2], and lifelong treatment with antibiotics to control the disease is not possible in food-producing animals [3]. Infection with MAP has been reported in ruminants worldwide. In the United Sates alone, most recent surveys suggest that more than 68% of dairy herds are infected with MAP, leading to considerable financial losses for producers [4].

Crohn’s disease (CD), a chronic, inflammatory gastrointestinal disease of humans, has many clinical and pathologic similarities to JD. The definitive cause of CD remains elusive. It is thought to result from a complex interaction of host susceptibility factors and an abnormally intense immune response to bacteria or other antigens in the intestines [5, 6]. Numerous studies have investigated the role of MAP in CD [7–8]. These studies have shown conclusively that MAP can be isolated from intestinal tissue of Crohn's patients (significantly more than controls), but the medical community still debates whether MAP causes the intestinal inflammation, or merely is able to colonize already-compromised intestinal tissues of afflicted individuals [9, 10].

The gastrointestinal microbiome is fundamental to the overall health and production performance of most mammals [11]. This diverse community of gastrointestinal bacteria exists in a delicate balance with the host- a balance that can be disrupted by changes in diet, antibiotic treatment, or infection with pathogenic bacteria [11]. Disruption of this balance, termed “dysbiosis” can result in gastrointestinal dysfunction, including inflammation of the intestinal lining [12]. In CD patients, studies of the gastrointestinal microbiome have shown evidence of dysbiosis, including a shift in the species of bacteria present and an overall reduced diversity of bacterial species [13, 14]. More specifically, those studies have shown a significant reduction in the proportion of bacteria belonging to the *Firmicutes* and *Bacteroidetes* phyla [13, 14]. It is speculated that this shift in enteric microbiota may contribute to the intestinal inflammation observed in these patients [14].

In cattle, the study of the gastrointestinal microbiota has mainly focused on the rumen, more specifically on the impact of different diets on its bacterial composition and the repercussion on animal productivity [15–17]. To the authors’ knowledge, the fecal microbial composition of MAP-infected cattle has not been studied. Given the similarities between the two syndromes, dysbiosis in cattle with JD, similar to that reported in patients with CD, is a reasonable expectation.

The objective of the study presented here was to investigate the diversity patterns of fecal bacterial populations in cattle infected with MAP, compared to those of uninfected control cattle, using phylogenomic analysis. We hypothesized that cattle naturally infected with MAP will have a reduction in gastrointestinal microbial biodiversity, when compared to the uninfected controls.

## Materials and Methods

### Fecal samples

Fecal samples were selected from the University of Pennsylvania Johne’s disease Laboratory’s fecal repository to include samples from 20 naturally infected, non-clinical MAP-positive cows (positive group); 25 MAP-negative herdmates (exposed group); and 25 MAP-negative cows from a MAP-free herd (negative group). All positive and exposed fecal samples were obtained from a single herd of 13,000 lactating cows. At the time of fecal sampling, MAP seroprevalence was 34.6% in the positive herd. All negative fecal samples were obtained from a MAP-negative herd of 300 lactating cows with no history of JD in the last 12 years. All fecal samples used were kept at -70°C prior to analysis. The status of the fecal samples selected had been pre-determined by standard mycobacterial culture on Herrold’s egg yolk medium [18], and confirmed as MAP using a commercially available RT-PCR kit (Vet Alert; Tetracore, Rockville, MD, USA).

### Sample Processing

Fecal samples were thawed and processed for genomic DNA extraction, using a commercially available DNA extraction kit (QIAamp DNA Stool Mini Kit; Qiagen, Valencia, CA, USA) as described elsewhere [19]. The extracted and purified DNA from the fecal samples was amplified for the V1-V2 hyper-variable regions of the 16S rDNA gene using barcoded 8F and 357R primers. Primer sequences and PCR conditions were similar to those described previously [20]. The amplicons were then bead purified using 1:1 Agentcourt AmPure XP beads (Beckman-Colter, Brea, CA, USA). The purified products from the fecal samples were pooled in equal concentration prior to pyrosequencing (GS FLX Titanium; Roche 454 Life Sciences, Branford, CT, USA).

### Sequence Analysis

The 16S rDNA sequences obtained were decoded and analyzed using the QIIME pipeline [21]. Reads were eliminated if they did not match the sample-specific barcode and amplified sequences were shorter than 200 bp or longer than 1000 bp, or contained a homopolymer sequences in excess of 6 bp. Operational taxonomy units (OTUs) were formed at 97% similarity using UCLUST [22] and representative sequences from each OTU were aligned to 16S rDNA reference sequences with PyNAST [23]. A phylogenetic tree was construed with FastTree [24]. Taxonomic assignments within the Greengenes taxonomy [25] were generated using RDP Classifier version 2.2 [26]. Alpha diversity of samples was calculated for each group, using Observed Species and Shannon diversity matrices. OTUs were rarified at a depth of 232 sequences, and subsampling was performed 10 times. The measured alpha diversities were compared between each group with a nonparametric two sample t-test using the default number of Monte Carlo permutations (999) [27]. The extent of relationship between bacterial communities was quantified using weighted and unweighted pairwise UniFrac distances [28]. Representative sequences of OTUs assigned to *Arthrobacter* were used for phylogenetic analysis. Only OTUs with a relative abundance of at least 0.2% were included for tree construction. A total of 78 sequences (16s sequences) related to *Arthrobacter* and *Mycobacterium* genera were obtained from NCBI nucleotide database [29]. Multiple sequence alignment was performed using Muscle [30]. Phylogenetic tree was reconstructed using FastTree with a generalized time-reversible (GTR) model [24].

### Statistical analysis

Statistical analysis and graphical presentation of data was performed using R program [31]. A non-parametric permutational multivariate ANOVA test [32], implemented in the vegan package for R [33], was conducted for each pair of bacterial communities, as measured by weighted UniFrac distance. To test for differences in taxon abundance, a generalized linear mixed model (GLMM) was constructed with the lme4 package for R [33]. The model used a binomial link function and included a random effect term for each animal. The input data for the mixed model consisted of a two-column matrix containing (in column 1) the number of reads assigned to the taxon, and (in column 2) the number of reads assigned to other taxa. Alpha diversity indices represented by Observed species and Shannon diversity calculated for each group were compared pairwise for positive vs. exposed and positive vs. negative using Wilcoxon rank sum test and the R package.

## Results

### Sequence information

Bacterial populations were surveyed by pyrosequencing fecal samples from seventy bacterial communities (70 animals) obtained from three sampling groups [negative = 25, exposed = 25, positive = 20]. Approximately 199,077 reads were obtained after quality control with an average of 2,843 reads per sample, three samples each from the positive and negative groups contains less than 1,000 reads per sample. Approximately 34,606 OTUs were produced by clustering at 97% sequence similarity and were assigned to 17 bacterial phyla and 117 known genera.

### Species richness and diversity

**Fig 1:**
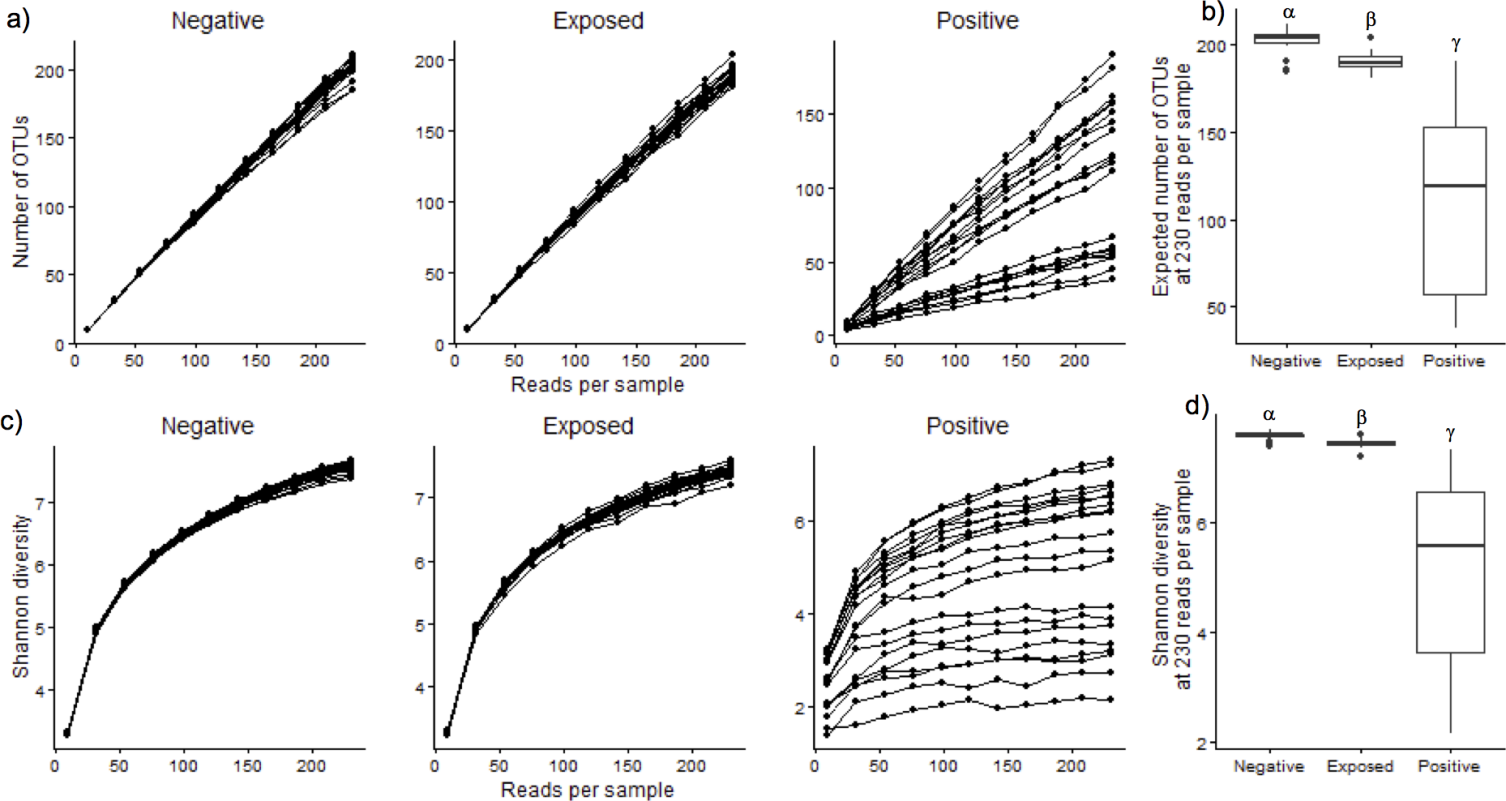
Richness and diversity metrics for fecal microbial communities of Negative, Exposed and Positive groups. a) Rarefaction curves showing the expected number of OTUs at different sequencing depth b) Boxplots showing expected number of OTUs at higher sequencing depth (232 reads per sample) c) Rarefraction curves showing the Shannon diversity values at different sequencing depth d) Boxplots showing Shannon diversity of OTUs at higher sequencing depth (232 reads per sample). α,β,γ indicates significant differences between the groups (P < 0.05;Wilcoxon test).

Species richness in the fecal microbiomes of negative, exposed and positive samples were compared using Rarefaction analysis (Fig 1). In the positive group, the majority of the samples had lower number OTUs compared to negative and exposed groups at different sequencing depths (Fig 1A). At higher sequencing depth (230 reads per sample), the expected number of OTUs was lower for positive group compared to negative and exposed groups (P <0.05, Wilcoxon rank sum; Fig 1B). Similarly, bacterial diversity using the Shannon Index showed marked differences between all 3 treatment groups (P <0.05, Wilcoxon rank sum; Fig 1C and 1D). Notably, a wide range of variation was observed among the positive samples for both richness and diversity indices. Similarly, bacterial diversity using the Shannon Index showed marked differences between positive vs. exposed and positive vs. negative groups (P <0.05, Wilcoxon rank sum; Fig 1C and 1D).

### Community Comparison

**Fig 2:**
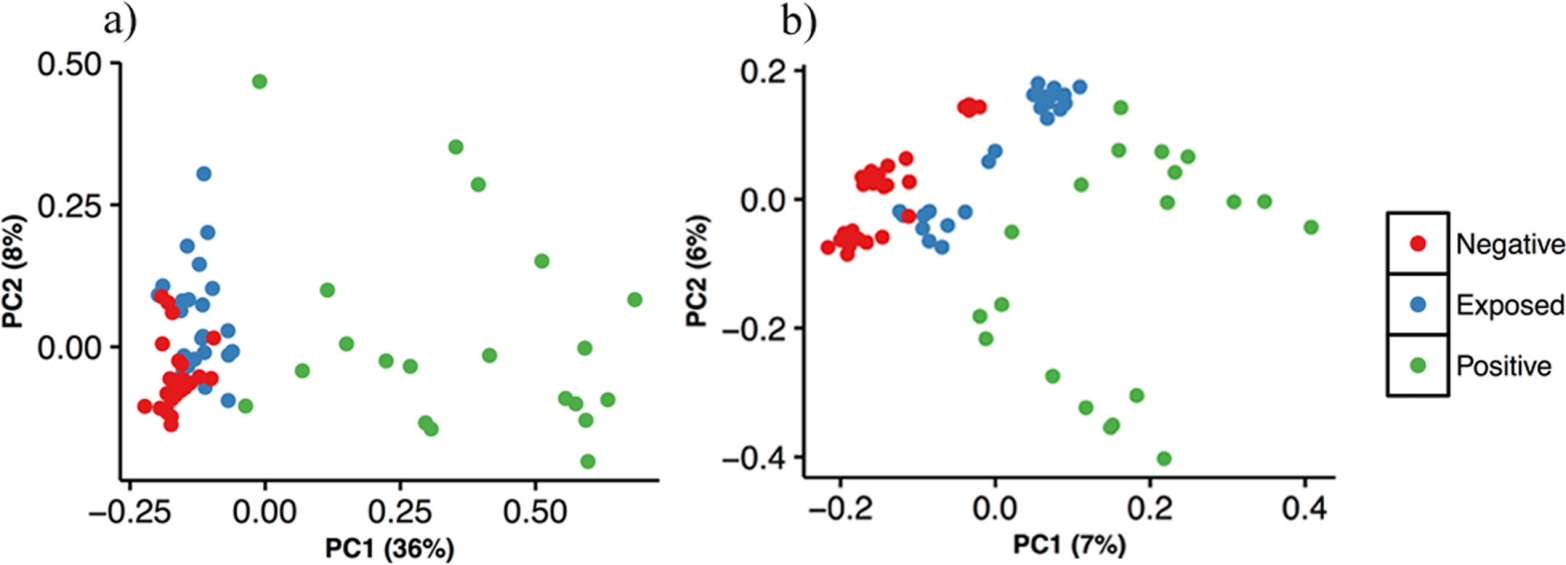
Comparison of bacterial community composition for negative, exposed and positive samples using principal coordinate analysis. a) Weighted UniFrac distances based on relative abundance of bacterial OTUs and b) Unweighted UniFrac distances based on presence/absence information of bacterial OTUs.

Weighted and unweighted UniFrac distances were calculated based on relative abundance and presence/absence information of OTUs in bacterial communities respectively. These distance matrices were visualized using principal coordinate analysis (PCoA) (Fig 2). Using the PERMANOVA test, we found that the bacterial communities were found to be different (P<0.001; PERMANOVA) among all three treatment groups. Differences among microbial communities of the three treatments explained 33% of variation in the weighted UniFrac model and 10% of variation in the unweighted UniFrac model (Table 1). Microbial community composition between individual treatment pairs (positive vs negative; positive vs exposed and exposed vs negative) were found to be different (P<0.001; PERMANOVA test; Table 1). However, differences in microbial community composition between negative and exposed was lower as explained by low variance in both weighted (10%) and unweighted (6%). Further, betadisper analysis confirmed that there were significant differences between positive/exposed and positive/negative treatment pairs (Table 1). To summarize, it was revealed from the unweighted Unifrac analysis that the MAP-positive group contained certain fecal microbiota which were not detected in the other two groups, implicating the proliferation of bacterial populations with incidence of MAP that are otherwise not detected in a healthy situation. Similarly, there were also substantial differences in the relative abundance of certain microbial populations that were detected in all three groups as depicted in the weighted Unifrac analysis. Finally, the betadisper test confirmed that fecal microbiota in the positive samples were less similar whereas both exposed and negative samples showed a greater degree of similarity.

**Table 1:** Permanova and betadisper analysis for the bacterial communities of bovine fecal samples collected from negative, exposed and positive groups.

	Permanova				Betadisper	
	Weighted		Unweighted		Weighted	Unweighted
	R^2^	*p* value	R^2^	*p* value	*p* value	*p* value
**Overall**	0.32917	0.001	0.10048	0.001	1.051e-10	2.415e-06
**Positive vs. Negative**	0.33809	0.001	0.09458	0.001	0.0000000^a^	0.0261055^a^
**Positive vs. Exposed**	0.30875	0.001	0.07507	0.001	0.0000000^a^	0.0000013^a^
**Negative vs. Exposed**	0.10226	0.001	0.06325	0.001	0.9921091^a^	0.0076192^a^

^a^ Quantified by Tukey post hoc test from betadisper output

### Taxonomic comparisons

**Fig 3:**
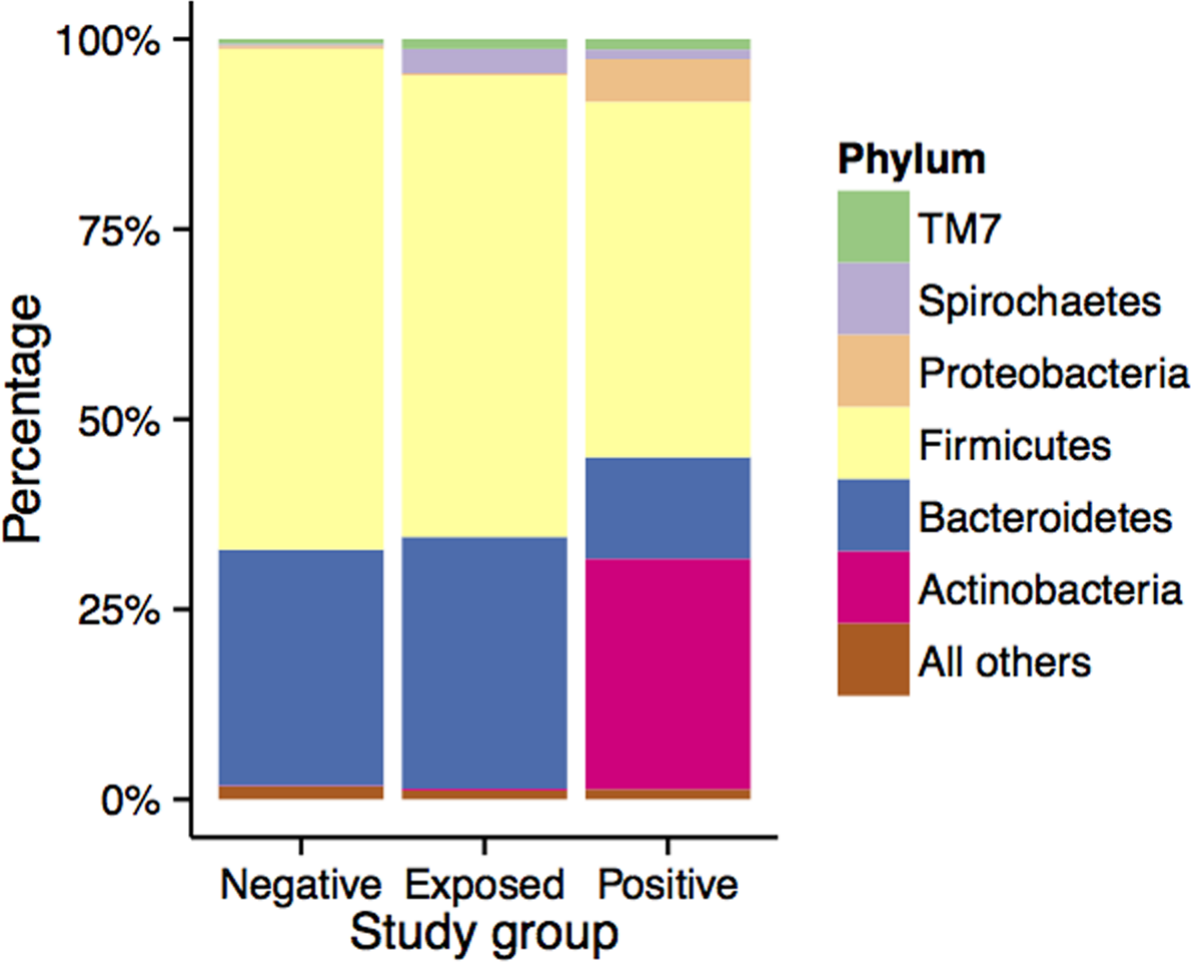
Stack bar plot showing the most abundant bacterial phyla in the bovine fecal samples collected from negative, exposed and positive groups.

**Fig 4:**
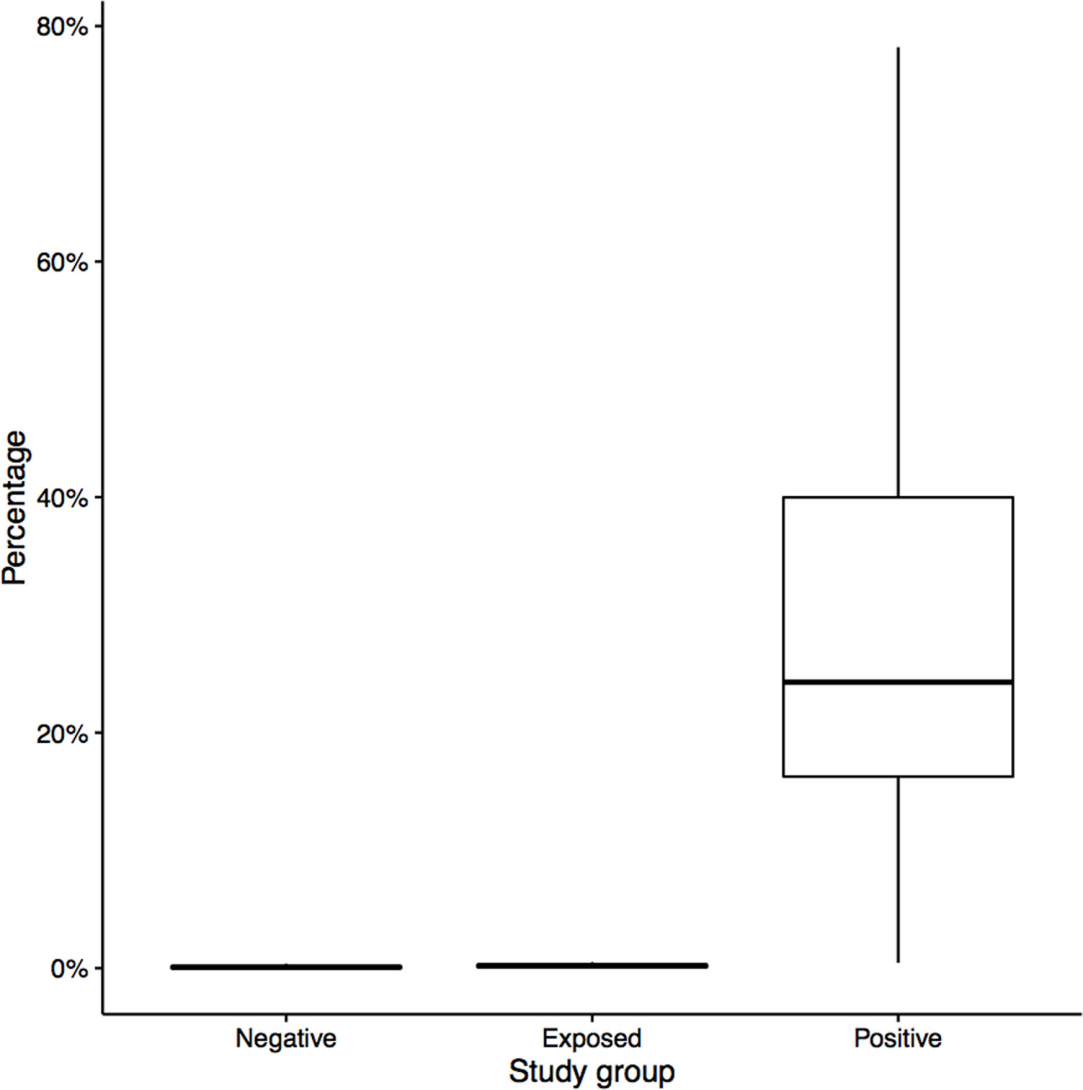
Box plot showing the abundance of *Actinobacteria* (%) detected in the bovine fecal samples collected from negative, exposed and positive groups. The top and bottom edges of each box correspond to the first and third quartiles of the data, respectively (25^th^ and 75th percentiles). The lines extend across the entire range of the data.

**Fig 5:**
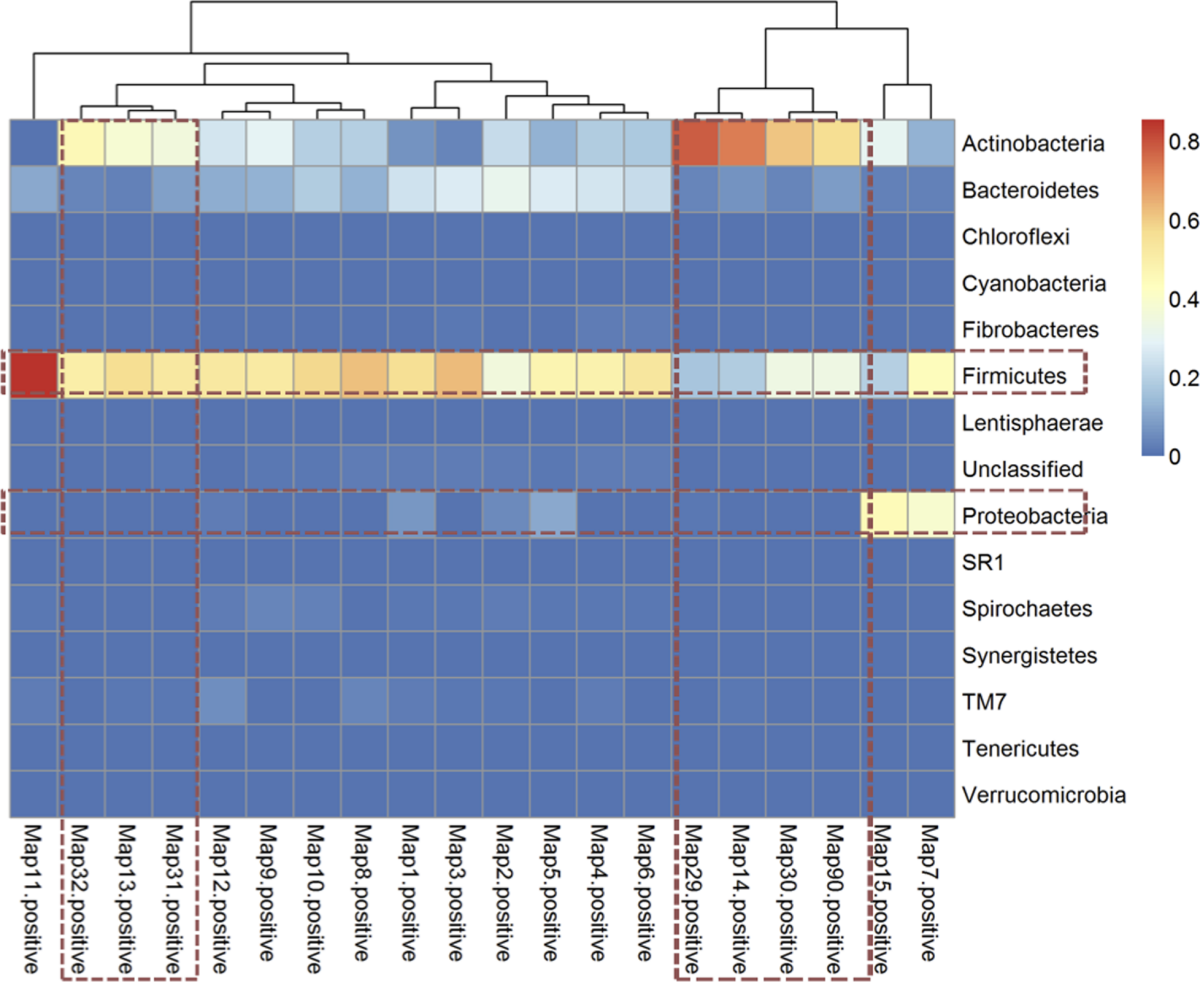
Thermal double dendogram showing abundance of phylum Proteobacteria and *Actinobacteria* compared to phylum *Firmicutes* and *Bacteroidetes* in fecal samples from the positive group.

Taxonomic assignment of the OTUs identified a total of 17 bacterial phyla across all samples. *Bacteroidetes* and *Firmicutes* constituted more than 95% of the total bacterial population in negative and exposed groups. However, in the positive group, lineages of *Bacteroidetes* and *Firmicutes* decreased with a concomitant increase in *Actinobacteria* and *Proteobacteria* (Fig 3). Further, the percent abundance of these bacterial phyla varied significantly (P < 0.001) among the individual samples in the positive group when compared to negative and exposed groups (Table 2; S1 Fig). For example, the phylum *Actinobacteria* was highly abundant (30% of the total bacteria) in the positive group compared to exposed and negative groups (0.1–0.2%; Fig 4). The extent of variation among the individual samples at the phylum level (Fig 5) demonstrates that the abundance of *Actinobacteria* and *Proteobacteria* increased at the expense of *Bacteriodetes* and *Firmicutes*. It appears that *Bacteroidetes* is the most vulnerable and was significantly reduced in all positive samples whereas reductions in *Firmicutes* depended on the abundance of *Actinobacteria*.

**Table 2:** Mean abundance (%) of bacterial phyla in the bovine fecal samples collected from negative, exposed and positive groups.

Phylum	Mean ± SEM^a^ (%)			Negative vs. Exposed^b^	Negative vs. Positive^b^
	Positive	Exposed	Negative		
**Actinobacteria**	30.321 ± 5.016	0.232 ± 0.030	0.109 ± 0.023	*	***
**Bacteroidetes**	13.358 ± 2.138	33.116 ± 1.065	31.024 ± 0.941	N.S.^c^	***
**Cyanobacteria**	0.240 ± 0.064	0.200 ± 0.061	0.414 ± 0.064	***	*
**Firmicutes**	46.738 ± 3.761	60.688 ± 1.063	65.877 ± 0.922	N.S.	***
**Proteobacteria**	5.604 ± 2.863	0.292 ± 0.039	0.409 ± 0.051	N.S.	N.S.
**Spirochaetes**	1.285 ± 0.277	3.197 ± 0.412	0.243 ± 0.032	***	***
**Tenericutes**	0.495 ± 0.075	0.690 ± 0.074	0.793 ± 0.074	N.S.	*
**TM7**	1.362 ± 0.302	1.296 ± 0.144	0.621 ± 0.097	***	**

^a^ Standard error of the mean

^b^ *p* values for the effect of study group ***: *P*<0.001; **: *P*<0.01; *: *P*<0.05

^c^ Not significant

At the family level (S1 Table), *Ruminococcaceae*, *Lachnospiraceae*, *Christenallaceae* decreased whereas *Enterococcaceae*, *Staphylococcaeae* and *Bacillaceae* (*Firmicutes*) increased in positive samples when compared to exposed and negative samples. Among the *Bacteroidetes* lineages, *Rikenellaceae*, *Prophyromonadaceae*, *Prevotellaceae* were greatly reduced (P < 0.001, GLMM) in positive samples. The predominance of *Micrococcaceae* of *Actinobacteria* lineage among the positive samples was noteworthy, as it was not detected in either the exposed or negative samples. The family *Moraxellaceae*, from the *Proteobacteria* group was found to be abundant in a few positive samples (P < 0.001, GLMM) while it was not detected in negative and exposed samples. On the contrary, there were several families (*Enterobacteriaceae*, *Succinivibrionaceae*, *Desulfovibrionaceae* and several unclassified members) among the *Proteobacteria* phylum, although contributing a very low abundance, were found to be higher (P < 0.01, GLMM) in the exposed and negative samples over positive samples.

**Fig 6:**
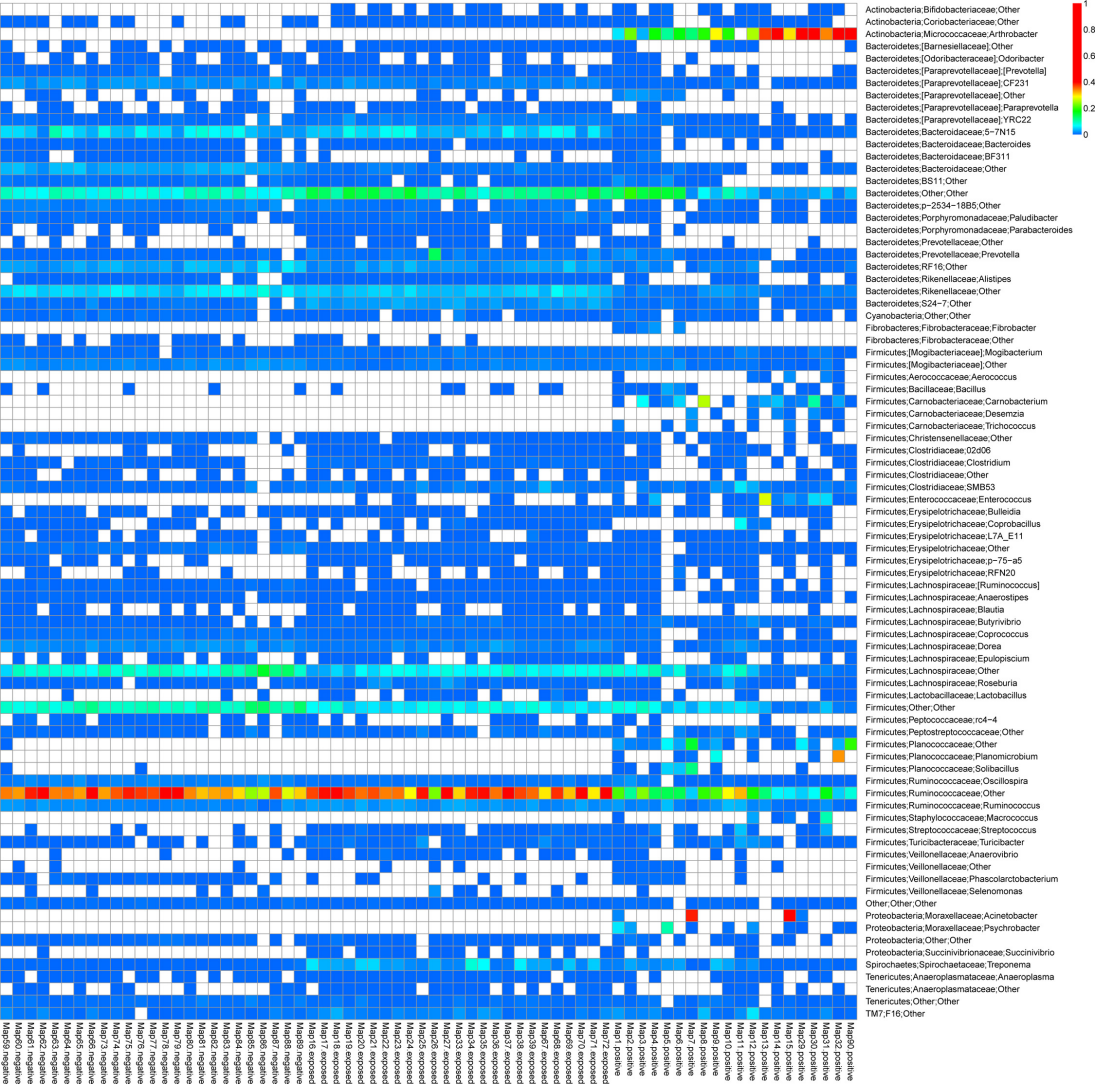
Thermal double dendrogram of the most abundant bacterial genera detected in the bovine fecal samples collected from negative, exposed and positive groups

At the genus level, approximately 117 genera were identified with 15 genera from *Bacteroidetes* and 20 genera from Firmicutes, with a proportion exceeding 1% in at least one sample. However, we found the predominance of a single genus, *Arthrobacter* from *Actinobacteria* across all positive samples, however this genus was not detected in exposed and negative groups. Among the *Bacteroidetes*, *Alistipes*, *Paraprevotella* and *Bacteroides* were reduced in abundance across all positive samples. Similarly, in phylum *Firmicutes*, genera *Clostridium* and *Ruminococcus* showed low abundance whereas *Bacillus* and *Enterococcus* were highly abundant in the positive group (Fig 6). Genera such as *Camobacterium*, *Desemzia* and *Trichococcus* (*Camobacteriaceae*) were only detected in positive group. Members of *Planococcaceae* (*Planomicrobium*, *Soilbacillus*) were in abundance in few samples of positive group, but were not detected in negative and exposed group.

**Fig 7:**
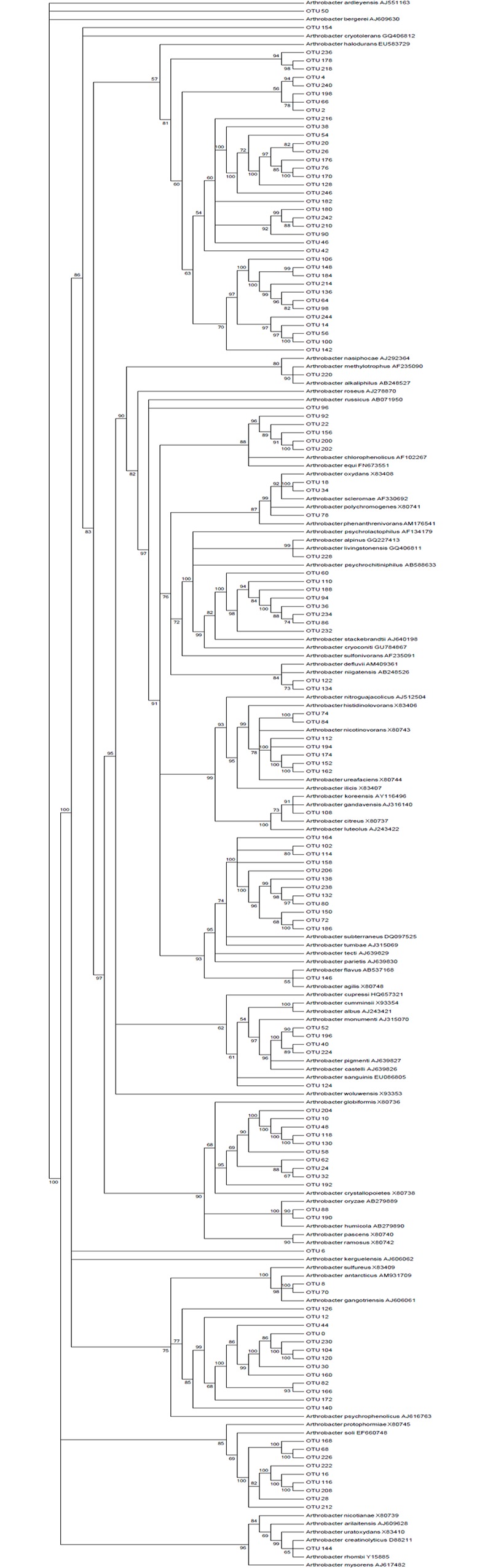
Phylogenetic tree of *Arthrobacter*. Phylogenetic tree of OTUs assigned to the genus *Arthrobacter*. The accession numbers are noted in parenthesis for each reference species.

To understand the extent of relationship between *Arthrobacter* partial 16S sequences identified in this study to that of cultured representative species from both *Arthrobacter* and MAP, a tree was constructed (Fig 7). A majority of *Arthrobacter* OTUs were closely related to *A*. *phenanthrenivorans Sphe3*, and a small proportion aligned to other species of Arthrobacter such as *A*. *globiformis*, *A*. *gangotriensis* and *A*. *nictonivorans*.

## Discussion

While the gastrointestinal tract hosts a wide range of bacterial species, four phyla are found to predominate in human and animal gut: *Firmicutes*, *Bacteroidetes*, *Proteobacteria* and *Actinobacteria* [34–35]. Among these four groups, Firmicutes alone account for nearly 64%, whereas *Bacteroidetes* account for approximately 23% of the normal human microbiota [36]. In healthy human subjects, the *Firmicutes* phylum is dominated by *Lachnospiraceae* members, whereas *Bacteroidales* members constitute the majority of the *Bacteroidetes* phylum [37]. However, in diseased conditions such as IBD and CD, commensal bacteria represented by *Bacteroidales* and *Lachnospiraceae* lineages decrease; while the *Bacillus* subgroup of *Firmicutes*, and members of *Proteobacteria* phylum increase [36]. The nature and extent of dysbiosis in enteric microbiota is variable with different clinical phenotypes of CD and IBD as one or more factors including disease location, environmental exposures, diets and host genetics can trigger the onset of these diseases [11]. And although the precise microbial species or metabolites involved are not completely understood, the endogenous intestinal microbiota is considered a major trigger of inflammation [38–40]. The first demonstrations of the involvement of intestinal microbiota in inflammatory bowel disease came from experiments showing that diversion of the fecal stream improved symptoms of CD, and that restoration of the fecal stream resulted in the induction of intestinal inflammation in those patients [41, 42]. It is now understood that this shift in fecal microbiota is associated with a decrease in bacteria with anti-inflammatory properties and an increase in bacteria with pro-inflammatory properties, all of which are believed to play a role in disease progression and maintenance [11].

In the study presented here, MAP-infected cattle had significant changes in their fecal microbiota when compared to MAP-negative herd-mates (exposed group), as well as MAP-negative cattle from a MAP-negative herd (negative group). Fecal samples of MAP-positive cattle had a significant reduction in phylum *Firmicutes* and *Bacteroidetes* when compared to cattle from the exposed and negative groups. The reduced proportion of healthy commensals such as *Lachnospiraceae* subgroup of *Firmicutes* and *Bacteroidetes* members was compensated by a higher representation of members of the *Proteobacteria* and *Actinobacteria* phyla, consistent with the dysbiosis observed in CD patients [13, 14]. Notably, *Arthrobacter* was found to be the predominant *Actinobacteria* in the positive group.

*Actinobacteria* include plant commensals (*Leifsonia* spp.), soil dwellers (*Streptomyces* spp.) and pathogens (*Corynebacterium* and *Mycobacterium*). The presence of genus *Arthrobacter* in anerobic environments such as the digestive tract of mammals has been reported, albeit in very low abundance (<0.1%) [43]. This is probably the first study reporting the prevalence of *Arthrobacter* in cattle feces infected with MAP. In the study reported here, the partial 16S sequences of *Arthrobacter* clustered with cultured *A*. *phenanthrenivorans* (Sphe 3). However, the similarity between *Arthrobacter* OTUs and *A*. *phenanthrenivorans* was variable, suggesting that these sequences may be novel. The relationship between *Arthrobacter* and MAP is unknown except for few earlier publications, suggesting that both bacteria require mycobactin for their growth [44, 45]. It was also reported that ISMap04, an insertion sequence that is found only in the genome of MAP strain K-10 has some similarity to *Arthrobacter* genus [46]. These evidences indicate an associative pattern between MAP and *Arthrobacter* and require further investigations to define the extent and role of such synergistic interactions and their role in the progression of Johne’s disease.

## Conclusions

In the study presented here, we investigated the intestinal microbiome of MAP-infected cattle, to determine if dysbiosis can be found in those animals. We demonstrated that fecal bacterial communities of MAP-positive cows varied significantly from those of cows from the exposed and negative groups. Furthermore, we demonstrated that bacterial communities within the exposed and negative groups were mostly homologous, whereas there was statistically significant greater variation between fecal samples in the MAP-positive cows. These findings are similar to the gastrointestinal dysbiosis demonstrated in patients suffering from various types of inflammatory bowel disease, including CD. Although the biological significance of the dysbiosis observed in the study presented here remains unclear, it is reasonable to speculate that it may play a role in the intestinal inflammation observed in the affected animals. A functional analysis of the bacterial communities within the positive samples would help understand the impact of such shifts on intestinal inflammation but is not included in this report.

These findings may provide potential targets for the treatment of MAP through probiotic agents designed to restore the microbiome to its proper balance and provide fodder for future longitudinal studies on the development of dysbiosis following experimental MAP infection, correlating these changes with parameters of intestinal inflammation.

## Supporting Information

S1 Fig Box plot showing differences in the bacterial phyla in the bovine fecal samples collected from negative, exposed and positive groups. (TIFF)

S1 Table Overall results of mean abundance (%) of bacterial phyla, families, and genuses in the bovine fecal samples collected from negative, exposed and positive groups. SEM: Standard error of the mean. *p* values for the effect of study group ***: *P*<0.001; **: *P*<0.01; *: *P*<0.05. NS: Not significant. NA: Not available.
(XLSX)
