# Supplementary figures and images for: Dysbiosis of the Fecal Microbiota in Cattle Infected with Mycobacterium avium subsp. paratuberculosis
Source: PLoS One. 2016 Aug 5;11(8):e0160353. doi: 10.1371/journal.pone.0160353 (PMC4975387; doi:10.1371/journal.pone.0160353)

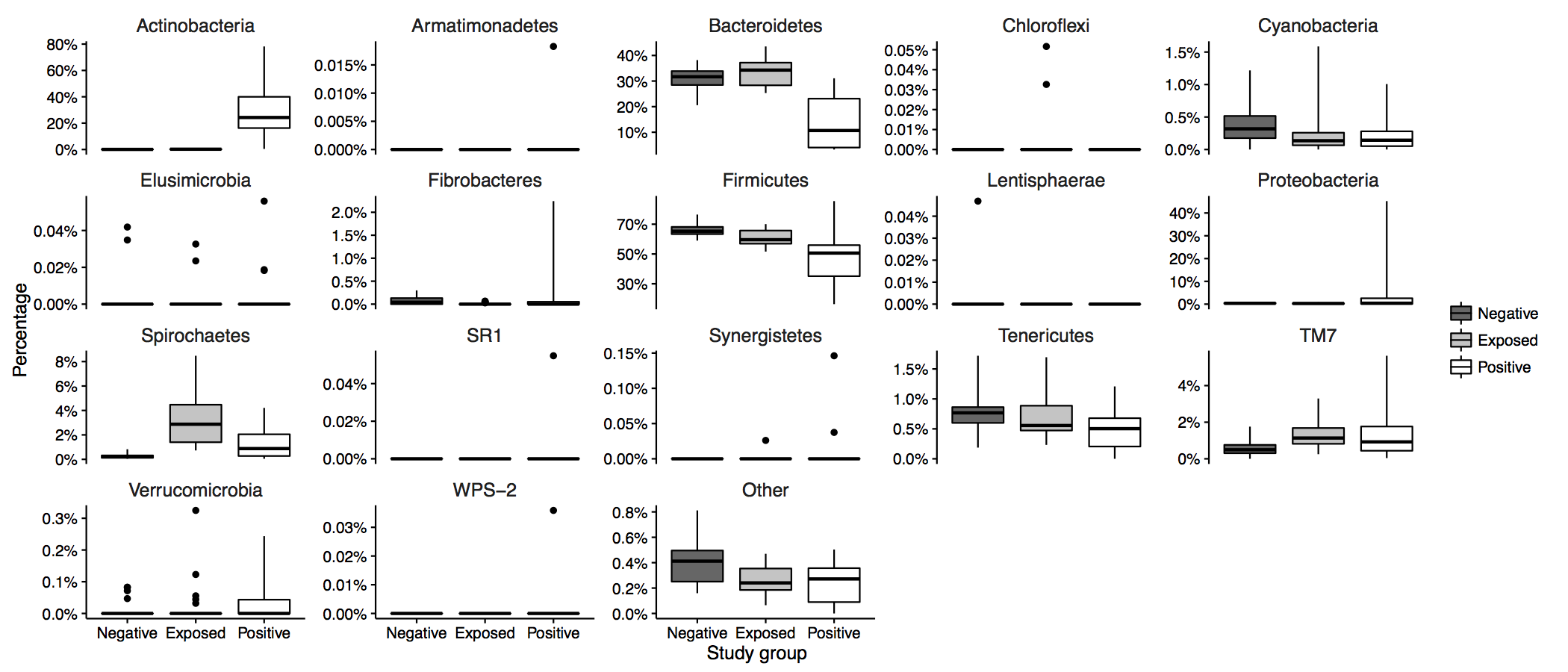

Supplement: S1 Fig — (TIFF) [file pone.0160353.s001.tiff]
